# Determinants of Sexual Satisfaction in Hemodialysis Patients: A Cross-Sectional Analysis with Healthy Controls

**DOI:** 10.3390/medicina61122171

**Published:** 2025-12-05

**Authors:** Leszek Sułkowski, Andrzej Matyja, Maciej Matyja

**Affiliations:** 1 Department of General Surgery, Regional Specialist Hospital, 42-218 Częstochowa, Poland; leszeksulkowski@szpitalparkitka.com.pl; 2 2nd Department of General Surgery, Jagiellonian University Medical College, 30-688 Kraków, Poland

**Keywords:** hemodialysis, sexual satisfaction, patient-reported outcomes, quality of life, dialysis adequacy, ultrafiltration, predialysis urea, vascular access, kidney transplantation, partner status

## Abstract

*Background and Objectives*: Sexual satisfaction is a distinct patient-centered outcome in hemodialysis. Yet its links to routine dialysis parameters remain unclear. To examine sexual satisfaction—assessed with the Sexual Satisfaction Scale (SSS)—in adults receiving maintenance hemodialysis in relation to demographic and dialysis parameters, and to compare SSS scores with healthy controls. *Materials and Methods*: Cross-sectional study of adults on maintenance hemodialysis (n = 72) and controls (n = 52). Sexual satisfaction was measured with the SSS (higher scores indicate greater dissatisfaction) among respondents with one primary partner. Demographic, clinical, and dialysis variables (shift, session duration, access, vintage, ultrafiltration, predialysis urea, Kt/V, urea reduction ratio), as well as transplant history/intent, were recorded. Group comparisons used t/Welch tests or ANOVA; correlations used Pearson’s r. *Results*: The SSS did not differ by sex, marital status, education, vascular access, dialysis shift, session duration, or adequacy indices. Prior kidney transplantation was associated with significantly lower SSS scores (mean 9.73 vs. 14.49; *p* = 0.0047), indicating better sexual satisfaction. *Conclusions*: Sexual dissatisfaction is common yet largely independent of routine demographic and dialysis metrics. Only previous kidney transplantation showed a significant association with sexual satisfaction in our cohort (*p* = 0.0047). Findings support a biopsychosocial, patient-centered approach and routine attention to sexual health in hemodialysis care.

## 1. Introduction

Sexual health is an essential, yet often neglected, dimension of quality of life in chronic kidney disease, particularly among individuals receiving maintenance hemodialysis. Across the chronic kidney disease spectrum, patients report high burdens of sexual symptoms that encompass diminished desire, arousal, and orgasmic difficulties, dyspareunia, and erectile dysfunction [1,2,3,4,5]. These disturbances arise from the combined effects of uremia, endocrine dysregulation, autonomic and vascular dysfunction, multimorbidity, medication effects, and psychosocial strain. In females on hemodialysis, qualitative work highlights pervasive body-image concerns (e.g., “bloated,” “deformed”), avoidance behaviors, and reduced sexual drive, while quantitative studies document deterioration in multiple sexual domains during dialysis with partial recovery after transplantation [2,3,4]. In males, erectile dysfunction is highly prevalent and frequently multifactorial, with contributions from hypogonadism, endothelial dysfunction, anemia, and mood disorders. Post-transplant cohorts often show clinically meaningful improvements, though residual dysfunction remains for many [6,7,8,9,10].

Beyond organ-specific physiology, the lived experience of hemodialysis also shapes intimacy. Fatigue, pain, cognitive strain, visual impairment, and restrictions in social participation have all been linked to dialysis and can erode relationship satisfaction and sexual well-being [11,12]. Social support consistently emerges as a protective factor, buffering psychological stressors and correlating with better patient-reported outcomes in hemodialysis populations [12]. At the same time, the literature cautions that “sexual dysfunction” (objective impairment on function scales) and “sexual satisfaction” (a subjective appraisal that integrates expectations, intimacy, and relationship quality) are related but distinct constructs [1,2,5]. For example, many female on hemodialysis meet dysfunction criteria primarily because of inactivity, yet a majority report being satisfied with their sexual lives [13].

Dialysis-related exposures could plausibly influence sexual satisfaction through multiple pathways. Treatment timing and logistics (e.g., shift assignment, session length) may alter fatigue patterns, sleep–wake cycles, caregiving responsibilities, and opportunities for intimacy [14,15]. Vascular access type can affect body image and the practicalities of sexual activity [3,5]. Volume status and ultrafiltration requirements intersect with cardiovascular stability, dyspnea, edema, and overall well-being—factors likely to modulate libido and satisfaction. Markers of dialysis “adequacy” such as Kt/V, the urea reduction ratio, and predialysis urea are routinely monitored; however, their relationship to sexual outcomes is mixed and may differ by sex, with some studies suggesting associations, especially in males, and others finding no clear links, particularly among females [16]. Treatment vintage (dialysis duration) is another candidate determinant, with reports that longer exposure may worsen sexual outcomes in some cohorts, although broader multidimensional effects of dialysis duration vary across studies [17,18]. Previous research indicates that sexual satisfaction may be influenced by clinical factors (e.g., diabetes, dialysis adequacy), psychosocial burden, and relationship dynamics; however, findings across studies remain inconsistent, with some reporting strong associations and others finding no significant links [2,5,14,15,16,17,18]. Diabetes mellitus, for example, exerts a clearly negative effect on sexual functions [19].

Renal transplantation is widely associated with improvements in multiple domains of sexual function and body image in both sexes, although not universally nor without residual deficits [2,6,7,8,9,10]. Nevertheless, whether the anticipation or willingness to undergo transplant relates to current sexual satisfaction during hemodialysis is uncertain and may be overshadowed by immediate symptom burden and relationship dynamics. Likewise, although hemodialysis changes the time burden and physiology of dialysis, randomized and observational data show heterogeneous effects on sexual activity and disease burden, with possible subgroup benefits (e.g., females, patients <60 years) rather than uniform gains [15]. Comparative studies show that both renal transplant recipients and hemodialysis patients display diminished erectile and intercourse-satisfaction functions but relatively preserved orgasmic ability [19]. Importantly, transplantation tends to normalize overall satisfaction and enhance sexual desire in nondiabetic men [19], while women after kidney transplantation report markedly better sexual relationships, function, and frequency than their hemodialysis counterparts [20].

Sexual dissatisfaction is a common, yet often overlooked, problem among patients receiving hemodialysis. Sexual function is one aspect of physical functioning, and sexual dysfunction—regardless of its etiology—may cause marked distress [21]. It is particularly frequent among women on hemodialysis and strongly related to increasing age, dyslipidemia, and depressive symptoms [21]. Despite the importance of these issues, prior research has concentrated mainly on physiological dysfunction rather than the subjective perception of satisfaction.

Despite the breadth of literature on sexual dysfunction in chronic kidney disease, several gaps persist. First, most studies emphasize function (e.g., erectile) rather than global sexual satisfaction, a patient-centered endpoint that integrates psychosocial context, partner dynamics, and expectation management [1,2,3,4,13]. Second, evidence connecting routine dialysis parameters—shift, session duration, access type, adequacy indices (Kt/V, urea reduction ratio, predialysis urea), ultrafiltration volume, and dialysis duration—to sexual satisfaction remains limited and inconsistent [5,14,15,16,17,18,22]. Third, few studies evaluate satisfaction in hemodialysis patients relative to healthy controls while considering partner status and gender distribution, which are key contextual modifiers of sexual well-being [12,13].

Against this background, we conducted a cross-sectional study of adult hemodialysis patients to examine sexual satisfaction—measured by the Sexual Satisfaction Scale (SSS) questionnaire—in relation to demographic, clinical, and dialysis-specific variables, and to compare SSS scores with those of healthy controls. By focusing on satisfaction rather than function alone, and by situating patient-reported outcomes alongside everyday dialysis metrics, we aim to inform pragmatic, multidisciplinary strategies for assessment and support in routine care. Given reports that sexual dysfunction affects up to 80% of women on hemodialysis [9,23,24,25], our analysis focused on satisfaction as a distinct, patient-reported outcome that may diverge from purely physiological function.

The primary aim of the study was to quantify the association between SSS scores and key dialysis parameters in hemodialysis patients—including dialysis shift, session duration, vascular access type, dialysis duration, ultrafiltration volume, predialysis urea, Kt/V, and urea reduction ratio. The secondary aim of the study was (i) to evaluate associations between SSS scores and demographic/sociobehavioral factors (age, sex, marital/partner status, education, and history of kidney transplantation, and willingness for future transplantation), and (ii) to compare SSS scores between hemodialysis patients and healthy controls after describing age and gender distributions in both groups.

## 2. Materials and Methods

### 2.1. Design, Settings, and Period of the Study

This cross-sectional study was conducted to evaluate determinants of sexual satisfaction among hemodialysis patients compared with healthy controls. The study design involved administering questionnaires, collecting demographic and clinical data, and comparing outcomes between patient and control groups. The study was carried out at the Dialysis Unit of the Regional Specialist Hospital in Częstochowa, Poland. Questionnaires were administered during mid-week dialysis sessions (Wednesday or Thursday) to minimize variability related to intradialytic timing and clinical status. A trained interviewer was available for clarification if needed; however, participants completed the questionnaires independently, without family or staff present.

### 2.2. Inclusion and Exclusion Criteria

Eligible participants were adults (≥18 years) diagnosed with end-stage renal disease and receiving maintenance hemodialysis. Patients were excluded if they had acute kidney injury, were undergoing peritoneal dialysis, were younger than 18 years, declined participation, or returned incomplete questionnaires. Seventy-two consecutive patients meeting these criteria were included. A control group of 52 healthy adults without a history of chronic kidney disease or dialysis was recruited, broadly matched to the patient group by age and sex distribution.

### 2.3. Data Collection and Procedure

Before enrollment, all participants were informed about the study aims and procedures and provided written informed consent. Participants were asked to reflect on their experiences over the previous four weeks. Demographic data (age, sex, marital status, education, and partner status) and clinical/dialysis-related variables were extracted from medical records and patient reports on the day of questionnaire administration. Dialysis variables included shift assignment (first, second, or third), vascular access type (arteriovenous fistula or central venous catheter), months on hemodialysis, session duration (hours), ultrafiltration volume per session (mL), predialysis urea concentration (mg/dL), and dialysis adequacy indices (Kt/V and urea reduction ratio). History of kidney transplantation (yes/no) and willingness to undergo future transplantation (yes/no) were also recorded.

### 2.4. Research Instrument

Sexual satisfaction was measured using the SSS questionnaire, adapted from the Sexual History Form and previously applied in chronic illness [26,27]. The SSS demonstrated good internal consistency in our sample (Cronbach’s α = 0.84). Prior studies have also confirmed acceptable reliability and content validity in chronic illness populations [26,27]. SSS was selected for its brevity, acceptability across sexes, feasibility in hemodialysis populations, and neutrality. The SSS includes four items addressing: satisfaction with physically expressed affection; variety of sexual activities; overall satisfaction with the sexual relationship; perceived partner satisfaction. Scores range from 4 to 24, with higher scores indicating greater dissatisfaction. To ensure relevance, all participants first answered a binary screening item: “Do you have a relationship with one primary partner?” Only respondents answering “Yes” completed the SSS items. Individuals without a primary partner did not complete the SSS but were retained for descriptive and comparative analyses where appropriate.

### 2.5. Ethical Considerations

The study protocol received approval from the local Bioethics Committee (K.B.Cz. 0014/2017). All procedures adhered to the Declaration of Helsinki. Written informed consent was obtained from all participants before inclusion.

### 2.6. Statistical Analysis

All analyses were performed using IBM SPSS Statistics for Windows, version 28.0 (IBM Corp., Armonk, NY, USA). Descriptive statistics were calculated for all baseline variables. Continuous variables were expressed as means with standard deviations (SD) and ranges, while categorical variables were summarized as frequencies and percentages. Normality of distribution for continuous variables—including SSS scores and dialysis parameters—was assessed using the Shapiro–Wilk test, along with visual inspection of histograms and Q–Q plots. Homogeneity of variances was tested using Levene’s test.

Comparisons between two independent groups (e.g., male vs. female, partnered vs. non-partnered, transplant vs. non-transplant) used the Student’s *t*-test when assumptions were met; otherwise, Welch’s *t*-test was applied. For comparisons involving more than two groups (e.g., dialysis shift, marital status, education level), one-way ANOVA or Welch’s ANOVA (if variances were unequal) was used. Associations between continuous variables (e.g., SSS score and age, dialysis duration, predialysis urea, ultrafiltration volume, Kt/V, urea reduction ratio) were examined using Pearson’s correlation for normally distributed variables or Spearman’s rank correlation otherwise. Correlation coefficients (r) were interpreted by strength and direction. Categorical group differences (e.g., gender distribution across groups) were evaluated with chi-squared tests; Fisher’s exact test was applied when expected cell counts were small. All tests were two-tailed, with statistical significance defined as *p* < 0.05. Exact *p*-values were reported for both significant and non-significant findings.

## 3. Results

### 3.1. Sexual Satisfaction and Age

We examined the association between sexual satisfaction and age (Table 1).

Among participants with a partner (n = 54), the mean age was 60.94 years (SD = 11.23, range 29–81), and the mean Sexual Satisfaction Scale (SSS) score was 14.0 (SD = 6.5, range 4–24). In participants without a partner (n = 18), the mean age was 67.44 years (SD = 14.77, range 28–84). The age difference between the two groups did not reach statistical significance (*p* = 0.100), although a tendency was observed for individuals without a partner to be older.

A weak positive correlation was identified between age and SSS scores (r = 0.249, *p* = 0.069), indicating that older age was slightly associated with lower sexual satisfaction. However, this relationship did not reach the threshold for statistical significance (*p* > 0.05).

### 3.2. Sexual Satisfaction and Gender

We analyzed the relationship between gender and sexual satisfaction, as assessed by the SSS (Table 2). The sample consisted of 34 men and 19 women.

Among participants with a partner, men (n = 34) reported a mean SSS score of 13.53 (SD = 7.74, range 4–24), whereas women (n = 20) reported a mean score of 10.79 (SD = 7.57, range 4–24). Among those without a partner, there were 10 men and 8 women.

The independent samples *t*-test indicated no statistically significant gender differences in SSS scores (*p* = 0.220). Although men tended to report somewhat higher levels of sexual satisfaction compared with women, the difference was small, and the distributions of scores overlapped considerably.

### 3.3. Sexual Satisfaction and Marital Status

We examined whether marital status was associated with differences in sexual satisfaction among dialysis patients, focusing on those in a relationship with a primary partner, and compared the distribution of marital status between patients with and without a partner (Table 3).

Among participants with a partner, the majority were married (n = 50), while only a small subgroup was single (n = 4); no widowed individuals reported having a partner. The mean SSS score among married participants was 12.52 (SD = 7.76, range 4–24), compared with 11.00 (SD = 9.02, range 4–24) among single participants. Among those without a partner, there were no married individuals, while 8 were single and 10 were widowed.

Analysis of variance (ANOVA) revealed no statistically significant differences in SSS scores across marital status categories (*p* = 0.711). Both married and single patients with a partner reported comparable levels of dissatisfaction, with only a slight, non-significant elevation in mean SSS scores among married individuals.

### 3.4. Sexual Satisfaction and Educational Level

We assessed the association between educational attainment and sexual satisfaction among dialysis patients with a partner, and compared the distribution of education levels between patients with and without a partner (Table 4).

Among participants with a partner, 19 had primary education, 21 had secondary education, and 13 had higher education. The corresponding mean SSS scores were 11.91 (SD = 9.53, range 4–24), 11.27 (SD = 5.08, range 7–24), and 15.75 (SD = 7.30, range 5–24), respectively. Among patients without a partner, 5 had primary education, 8 had secondary education, and 4 had higher education.

Analysis of variance (ANOVA) indicated no statistically significant differences in SSS scores across education levels (*p* = 0.633). Although patients with higher education reported somewhat higher SSS scores—suggesting greater dissatisfaction—this effect was modest and accompanied by wide variability.

### 3.5. Sexual Satisfaction and Dialysis Duration

We examined whether dialysis duration was associated with sexual satisfaction and whether treatment duration differed between patients with and without a primary partner (Table 5).

Among participants with a partner (n = 54), the mean duration of dialysis was 60.24 months (SD = 71.94, range 1–317), compared with 28.00 months (SD = 28.73, range 2–108) among those without a partner (n = 18). Welch’s *t*-test demonstrated a statistically significant difference in dialysis duration between the two groups (*p* = 0.011), indicating that patients in relationships had, on average, been undergoing dialysis for a longer period.

The mean SSS score among patients with a partner was 14.0 (SD = 6.5, range 4–24). The correlation between dialysis duration and SSS score was weak and negative (r = –0.11, *p* = 0.44), suggesting a slight tendency for longer dialysis duration to be associated with lower sexual satisfaction. However, this relationship was not statistically significant

### 3.6. Sexual Satisfaction and Vascular Access for Dialysis

We investigated whether the type of vascular access for dialysis was associated with sexual satisfaction among patients in a relationship with a primary partner (Table 6).

Among partnered participants, 46 used an arteriovenous fistula, and 8 had a central venous catheter. The mean SSS score was 11.92 (SD = 7.46, range 4–24) in the arteriovenous fistula group and 11.88 (SD = 6.47, range 5–24) in the central venous catheter group. Among those without a partner, 15 used arteriovenous fistulas, and 3 had a central venous catheter.

Welch’s *t*-test indicated no statistically significant difference in SSS scores between patients with arteriovenous fistula and those with central venous catheter access (*p* = 0.991). Both groups demonstrated wide variability in reported sexual satisfaction, suggesting that the type of vascular access does not substantially influence SSS scores in this population.

### 3.7. Sexual Satisfaction and Dialysis Shift

We investigated whether the timing of dialysis sessions (first, second, or third shift) was associated with sexual satisfaction among patients in a relationship, and compared dialysis shift distribution between those with and without a partner (Table 7).

Among partnered participants, 19 were dialyzed during the first shift, 20 during the second, and 15 during the third. The corresponding mean SSS scores were 12.95 (SD = 9.19, range 4–24), 12.25 (SD = 6.78, range 4–24), and 11.93 (SD = 7.57, range 4–24). Among patients without a partner, 8 were in the first shift, 7 in the second, and 3 in the third.

ANOVA analysis revealed no statistically significant differences in SSS scores between dialysis shifts (*p* = 0.928). Mean levels of sexual dissatisfaction were comparable across all three shifts, although greater variability was observed among patients dialyzed in the first shift

### 3.8. Sexual Satisfaction and Duration of Dialysis Session

We evaluated whether hemodialysis session duration was associated with sexual satisfaction and compared session length between patients with and without a primary partner (Table 8).

Among partnered participants (n = 54), the mean session duration was 4.04 h (SD = 0.44, range 2.0–5.0), compared with 3.92 h (SD = 0.46, range 3.0–5.0) among those without a partner (n = 18). The difference in session length between groups was not statistically significant (*p* = 0.331).

The mean SSS score among patients with a partner was 14.0 (SD = 6.5, range 4–24). Within this group, dialysis session duration was not significantly correlated with SSS scores (r = 0.047, *p* = 0.739). These findings indicate that session duration was not associated with sexual satisfaction in this cohort.

### 3.9. Sexual Satisfaction and a History of Kidney Transplant

We compared sexual satisfaction between dialysis patients in relationships with and without a history of kidney transplantation (Table 9).

Among partnered participants, 39 reported no history of transplantation, and 15 reported a previous transplant. The mean SSS score was 14.49 (SD = 6.65, range 4–24) in patients without a transplant history and 9.73 (SD = 6.72, range 4–24) in those with a transplant history. Among patients without a partner, 17 had no transplant history, and 1 reported a prior transplant.

A Welch’s *t*-test demonstrated a statistically significant difference in SSS scores between the two groups (*p* = 0.0047), with patients without a history of transplantation reporting higher mean SSS scores, consistent with greater sexual dissatisfaction.

### 3.10. Sexual Satisfaction and Future Transplant Desire

We examined whether willingness to undergo kidney transplantation in the future was associated with sexual satisfaction among dialysis patients in relationships (Table 10).

Among partnered participants, 49 reported no desire for transplantation, and 5 expressed willingness to receive a transplant. The mean SSS score was 14.14 (SD = 7.02, range 4–24) in patients unwilling to undergo transplantation and 11.50 (SD = 4.93, range 6–18) in those willing. Among participants without a partner, 16 reported no willingness, and 2 expressed willingness to undergo transplantation.

Although patients who were open to future transplantation reported slightly lower mean SSS scores, the difference between groups was not statistically significant (*p* = 0.587). These findings indicate that willingness to undergo transplantation was not associated with sexual satisfaction in this cohort.

### 3.11. Sexual Satisfaction and Urea Levels

We evaluated whether pre-dialysis urea levels were associated with sexual satisfaction and whether urea levels differed between patients with and without a primary partner (Table 11).

Among patients with a partner (n = 54), the mean pre-dialysis urea concentration was 120.89 mg/dL (SD = 37.42, range 72–202), compared with 136.19 mg/dL (SD = 34.51, range 72–191) in those without a partner (n = 18). Welch’s *t*-test indicated that this difference was not statistically significant (*p* = 0.138).

The mean SSS score among partnered patients was 14.0 (SD = 6.5, range 4–24). Correlation analysis revealed a weak positive association between pre-dialysis urea levels and SSS scores (r = 0.185, *p* = 0.189), suggesting a tendency for higher urea levels to be linked with greater sexual dissatisfaction. However, this relationship did not reach statistical significance.

### 3.12. Sexual Satisfaction and Ultrafiltration Volume

We examined whether ultrafiltration volume was associated with sexual satisfaction and compared ultrafiltration levels between patients with and without a primary partner (Table 12).

Among partnered patients (n = 54), the mean ultrafiltration volume was 2438 mL (SD = 947, range 300–4200), compared with 1761 mL (SD = 712, range 300–3000) in those without a partner (n = 18). Welch’s *t*-test showed this difference to be statistically significant (*p* = 0.0029), indicating that patients in relationships underwent greater fluid removal during dialysis sessions.

The mean SSS score among partnered patients was 14.0 (SD = 6.5, range 4–24). Correlation analysis revealed a weak negative association between SSS scores and ultrafiltration volume (r = –0.183, *p* = 0.195). This suggests that higher ultrafiltration may be modestly linked with lower sexual dissatisfaction, although the relationship was not statistically significant.

### 3.13. Sexual Satisfaction and Kt/V

We assessed the association between dialysis adequacy, expressed as Kt/V, and sexual satisfaction among patients in relationships, and compared Kt/V values between patients with and without a partner (Table 13).

Among partnered patients (n = 54), the mean Kt/V was 1.297 (SD = 0.264, range 0.661–1.895), with a mean SSS score of 14.0 (SD = 6.5, range 4–24). In patients without a partner (n = 18), the mean Kt/V was 1.343 (SD = 0.489, range 0.818–1.572). Welch’s *t*-test showed no statistically significant difference in dialysis adequacy between partnered and non-partnered patients (*p* = 0.702).

Among patients in relationships, correlation analysis demonstrated a weak negative association between Kt/V and SSS scores (r = –0.240, *p* = 0.195), suggesting that higher dialysis adequacy may be modestly associated with lower sexual dissatisfaction. However, this relationship was not statistically significant.

### 3.14. Sexual Satisfaction and Urea Reduction Ratio

We evaluated the association between sexual satisfaction, measured by the Sexual Satisfaction Scale (SSS), and dialysis adequacy as assessed by the urea reduction ratio (Table 14).

Among patients with a partner (n = 54), the mean urea reduction ratio was 0.659 (SD = 0.075, range 0.420–0.802), with a mean SSS score of 14.0 (SD = 6.5, range 4–24). In patients without a partner (n = 18), the mean urea reduction ratio was 0.666 (SD = 0.089, range 0.507–0.904). Welch’s *t*-test demonstrated no statistically significant difference in urea reduction ratio between partnered and non-partnered patients (*p* = 0.76).

Among those in relationships, the correlation between SSS scores and urea reduction ratio was weakly negative (r = –0.14, *p* = 0.25), suggesting that higher dialysis adequacy may be modestly associated with lower sexual dissatisfaction. However, this relationship did not reach statistical significance.

### 3.15. Sexual Satisfaction in Dialysis Patients and Healthy Controls

We compared demographic characteristics and sexual satisfaction scores between dialysis patients and healthy controls (Table 15 and Table 16).

The dialysis group included 72 individuals (mean age 62.3 years, SD = 12.6, range 28–84), while the control group consisted of 52 individuals (mean age 60.6 years, SD = 13.4, range 36–86). The difference in age distribution between the two groups was not statistically significant (*p* = 0.268). Gender distribution was also similar, with 44 males and 28 females in the dialysis group and 32 males and 20 females in the control group (*p* = 1.00).

The mean SSS score among dialysis patients was 9.18 (SD = 8.64), compared with 9.26 (SD = 6.25) in healthy controls. Welch’s *t*-test indicated no statistically significant difference in sexual satisfaction between the groups (*p* = 0.951).

Subgroup analysis by gender showed that male dialysis patients had a mean SSS score of 10.45 (SD = 8.95), compared with 8.84 (SD = 5.72) in male controls (*p* = 0.395). Female dialysis patients had a mean score of 7.50 (SD = 7.92), compared with 9.44 (SD = 6.82) in female controls (*p* = 0.318). These results demonstrate that sexual satisfaction, as measured by the SSS, did not differ significantly between dialysis patients and healthy controls in either males or females.

## 4. Discussion

This study investigated sexual satisfaction among hemodialysis patients using the SSS questionnaire, examining demographic, clinical, and treatment-related factors, as well as comparisons with healthy controls. Overall, sexual dissatisfaction was prevalent, with mean SSS scores indicating moderate dissatisfaction across most subgroups. Patients without a partner tended to be older than those with a partner, though the difference was not statistically significant. A weak, non-significant positive correlation was observed between age and SSS scores, suggesting a trend toward greater dissatisfaction with increasing age. Sexual satisfaction did not differ significantly across dialysis shifts, session duration, or dialysis adequacy measures (Kt/V, urea reduction ratio, urea levels). Similarly, vascular access type (arteriovenous fistula vs. central venous catheter) was not associated with SSS outcomes. Dialysis duration was significantly longer in patients with a partner compared to those without, although no significant correlation was found between dialysis duration and SSS scores. Patients with partners also had significantly higher ultrafiltration volumes, but this did not correlate with dissatisfaction. Marital status and education level were not significantly associated with SSS outcomes. Male patients tended to report slightly higher dissatisfaction than females, but the difference was not statistically significant. Patients with a history of kidney transplantation reported significantly lower SSS scores (indicating less dissatisfaction) compared with those without a transplant. In contrast, future transplant desire was not associated with statistically significant differences in SSS scores. Age and gender distribution were comparable between dialysis patients and healthy controls. Mean SSS scores were almost identical in the two groups, with no statistically significant differences observed overall or when stratified by gender.

Most demographic and dialysis-related factors showed no significant association with sexual satisfaction, except for dialysis duration and ultrafiltration volume (partnered vs. non-partnered patients) and a history of kidney transplantation, which was associated with significantly lower dissatisfaction.

### 4.1. Sexual Satisfaction and Age

In our cohort of dialysis patients, those without a primary partner tended to be older (67.44 ± 14.77 vs. 60.94 ± 11.23 years), although this difference did not reach statistical significance. Among partnered patients, sexual dissatisfaction as measured by the SSS showed a weak, non-significant tendency to increase with advancing age. These findings suggest possible age-related patterns, but the associations were not statistically conclusive and require cautious interpretation.

Age is widely recognized as a major determinant of multiple health domains in hemodialysis, including fatigue, cognitive function, pain, and visual impairment [11,17]. However, in our partnered cohort, the direct relationship between age and sexual satisfaction was not significant. This may indicate that for individuals in established relationships, sexual satisfaction is a complex, multifactorial construct influenced not only by physiological aging but also by psychological, emotional, and social factors that can mitigate the direct effects of age [12]. Previous research similarly suggests that younger patients often demonstrate more favorable overall health outcomes, which may indirectly support intimacy and satisfaction [17].

The absence of a strong age-related decline in sexual satisfaction in our study warrants further investigation with larger samples to uncover subtler associations and potential adaptive mechanisms. Our observations—namely, a non-significant trend toward greater dissatisfaction with advancing age among partnered patients and a tendency for unpartnered patients to be older—are consistent with broader CKD guidance and syntheses emphasizing the cumulative effects of aging and comorbidity on sexual health across the dialysis–transplant continuum. At the same time, they underscore that age alone rarely explains patient-reported sexual satisfaction in isolation [28,29]. Notably, a recent meta-analysis in women with end-stage renal disease demonstrated a very high prevalence of sexual dysfunction, with older age and menopause as key correlates, but also highlighted the roles of adaptation, intimacy patterns, and expectations [29]. These findings align with the conceptual separation between physiologic dysfunction and subjective satisfaction that has been emphasized in consensus statements and systematic reviews.

### 4.2. Sexual Satisfaction and Gender

In our study, no statistically significant difference in sexual satisfaction (SSS scores) was observed between male and female dialysis patients in primary relationships. This suggests that gender alone may not be a determining factor for sexual satisfaction in this cohort. This finding is noteworthy given the consistently high prevalence of sexual dysfunction in end-stage renal disease, with erectile dysfunction reported in up to 75% of males and diverse sexual difficulties—including diminished desire, arousal disturbances, dyspareunia, and orgasmic problems—affecting 30–80% of females [1,2,3,4]. Female patients are additionally vulnerable to body image concerns and practical barriers to intimacy related to vascular access [3,5]. Female hemodialysis patients in our cohort reported lower satisfaction scores than men, but the difference was not statistically significant. Prior work demonstrates that sexual-function scores are severely impaired among women with end-stage renal disease and relate strongly to comorbidity and mood disorders [9,23,25]. Many women undergoing hemodialysis experience some degree of sexual dysfunction, yet a substantial number still report moderate satisfaction with their sexual lives [13,30]. These findings are consistent with broader evidence that sexual dysfunction is highly prevalent in both men and women with end-stage kidney disease and often coexists with anxiety, depression, and reduced self-esteem [23,25]. Although physiologic dysfunction is frequent, many patients report preserved or even normal satisfaction, suggesting a dissociation between sexual function and subjective well-being [13,28,30]. This paradox underscores the need to consider both physiological and psychosocial dimensions of sexual health.

Importantly, sexual dysfunction (objective physiologic impairment) and sexual satisfaction (subjective appraisal) are related but distinct constructs. Mor et al. showed that many women on hemodialysis fulfilled criteria for sexual dysfunction, often due to sexual inactivity, yet still reported satisfaction with their sexual lives [13]. Our results, which emphasize subjective satisfaction within relationships despite documented dysfunction, likely reflect adaptation, recalibrated expectations, and the supportive role of intimate partnerships. This interpretation underscores the importance of individualized, gender-sensitive assessment and tailored psychosocial support [1,13].

The absence of gender differences in our cohort is further consistent with reviews and cohort studies indicating that, although men and women on dialysis frequently experience sex-specific dysfunctions (e.g., erectile dysfunction in males; reduced desire, arousal difficulties, and dyspareunia in females), subjective satisfaction may remain relatively preserved in a substantial subset of patients, particularly when coping mechanisms and stable relationships are present [13,23,28]. Large syntheses in female populations, both on dialysis and post-transplant, confirm high dysfunction prevalence but also demonstrate improvements following transplantation. Importantly, these studies emphasize that satisfaction scores often decouple from measures of activity or frequency [28,29]. This helps reconcile our null gender finding with the well-documented sex-specific patterns of physiologic dysfunction and highlights the multifactorial nature of sexual well-being in dialysis patients.

Our findings support the concept that sexual satisfaction and sexual function are related but not equivalent. Sexual dysfunction may affect up to 80% of women with chronic kidney disease [23,30], yet many remain satisfied with their sexual lives [13,30]. Measurement instruments such as the Female Sexual Function Index can overestimate dysfunction when administered to sexually inactive women [30]. Therefore, subjective satisfaction scales like the SSS offer complementary insight. This distinction is essential for developing realistic, patient-centered interventions [31].

### 4.3. Sexual Satisfaction and Marital Status

In our study, among patients in a primary relationship, no significant differences in sexual satisfaction (SSS scores) were observed between married individuals and those in other committed partnerships. This suggests that marital status as a formal designation may not significantly influence sexual satisfaction in this hemodialysis population. Small group sizes, particularly for non-married partnered respondents, limit interpretability, but the overall pattern indicates that the presence of a relationship itself, rather than its legal status, is more relevant for sexual well-being.

Prior research highlights the importance of social support often provided by a spouse or partner in improving the quality of life for dialysis patients [12]. Indeed, other studies have shown that married individuals may report higher levels of emotional, informational, and affectionate support, which could indirectly benefit sexual well-being even if marital status itself shows no direct association [12]. Our findings, therefore, align with broader evidence suggesting that the quality and presence of supportive relationships, rather than legal marital status alone, drive better patient-reported outcomes in dialysis, including emotional health and coping [12,32].

Psychosocial frameworks in chronic kidney disease further emphasize that sexual well-being is shaped by factors such as dyadic adjustment, communication, and body image, which extend beyond civil status [32]. Thus, our results are consistent with a model in which supportive relational dynamics, rather than marital designation, serve as key determinants of sexual satisfaction in this population.

### 4.4. Sexual Satisfaction and Educational Level

This study evaluated the influence of educational attainment on sexual satisfaction among dialysis patients with a partner. Although mean SSS scores varied by education group—from 11.27 in patients with secondary education to 15.75 in those with higher education—these differences did not reach statistical significance. The distribution of education levels differed slightly between partnered and non-partnered patients, with vocational or secondary education most common among those with a partner, yet these differences did not translate into variations in reported sexual satisfaction.

Our investigation therefore revealed no statistically significant association between educational attainment and SSS. While lower education is often linked with poorer outcomes in several quality-of-life domains among dialysis patients, including higher stress levels and worse general health [12,22], such effects did not extend to sexual satisfaction in our cohort. This underscores the multifactorial nature of sexual satisfaction in chronic illness, which is likely more strongly shaped by psychosocial and clinical variables than by education level alone.

Consistent with this interpretation, recent syntheses and consensus statements do not identify education as an independent determinant of sexual satisfaction once mediating influences such as relationship quality, psychological well-being, and comorbidity are considered [12,22]. Our findings therefore reinforce the need to examine broader psychosocial and clinical contributors when evaluating sexual well-being in dialysis populations, rather than attributing differences to educational attainment *per se*.

### 4.5. Sexual Satisfaction and Dialysis Duration

In our cohort, patients with a primary partner had a significantly longer dialysis tenure compared to those without a partner. This difference may reflect that maintaining relationships could be more feasible with longer dialysis experience, or that established relationships foster resilience over time. However, within the subgroup of partnered patients, dialysis duration did not significantly correlate with SSS, suggesting that extended treatment duration alone does not directly influence sexual satisfaction.

The literature offers mixed evidence regarding the relationship between dialysis duration and sexual function. Some reports describe worsening satisfaction with longer dialysis vintage [2,18], while others demonstrate stability over time [2,17,18,33]. Such discrepancies may reflect differences in study populations, cultural context, and measurement instruments. Potential mediators—including fatigue, depression, and symptom burden—are frequently cited as key drivers of declining sexual function [17,18]. At the same time, patient-reported outcomes research suggests that coping strategies and relational adaptation can buffer the negative effects of prolonged dialysis on subjective satisfaction, even when physiologic dysfunction persists [18,33].

Taken together, our findings indicate that while dialysis duration differs significantly between partnered and unpartnered patients, sexual satisfaction in partnered patients appears to be shaped less by treatment duration itself than by psychosocial and clinical mediators. Longitudinal studies are needed to clarify temporal dynamics and to disentangle the relative contributions of resilience, adaptation, and comorbid symptomatology to sexual well-being in this population.

### 4.6. Sexual Satisfaction and Vascular Access for Dialysis

We observed no meaningful differences in SSS between patients with arteriovenous fistulae and central venous catheters suggesting that vascular access type does not meaningfully affect reported sexual satisfaction in partnered dialysis patients. While access may influence body image and intimacy logistics, global satisfaction appears to be shaped predominantly by broader biopsychosocial factors rather than access type per se [21,23,30,31]. This finding implies that clinical decisions regarding vascular access may not directly influence sexual well-being, at least as assessed by the SSS.

Although vascular access is central to daily life in hemodialysis and can influence body image, comfort, and the logistics of intimacy—particularly among female patients—its direct association with global sexual satisfaction appears limited in our data [3,5]. Our findings, therefore, suggest that other physiological, psychological, and relational determinants likely exert greater influence. This interpretation is supported by qualitative and psychometric research showing that vascular access can shape intimacy and body image, but that overall satisfaction is more strongly linked to psychosocial factors [3,5,32].

Accordingly, our null finding is consistent with a person-centered model of care, in which communication, partner support, and effective management of symptoms outweigh the impact of access type itself on sexual well-being.

### 4.7. Sexual Satisfaction and Dialysis Shift

In this analysis of 72 dialysis patients, no statistically significant relationship was found between dialysis shift (1st, 2nd, or 3rd) and sexual satisfaction as measured by the SSS. Although mean SSS scores were slightly higher in the first shift, the differences were not statistically meaningful, and the timing of dialysis sessions did not emerge as a determinant of subjective sexual satisfaction in this cohort. A slightly higher representation of non-partnered patients in earlier shifts did not translate into measurable differences in satisfaction.

Although some studies suggest that selected quality-of-life domains may vary by dialysis scheduling, including sleep patterns and daytime functioning, evidence regarding sexual outcomes remains sparse and inconsistent [12,14,22]. Our findings, therefore, imply that dialysis scheduling alone is unlikely to have a meaningful effect on sexual well-being. Instead, sexual satisfaction in hemodialysis patients is likely more strongly influenced by psychosocial and clinical determinants such as fatigue, sleep disruption, mood disorders, and relational context. Interventions aiming to improve sexual satisfaction should therefore prioritize addressing these broader factors rather than focusing solely on session timing.

### 4.8. Sexual Satisfaction and Duration of Dialysis Session

In our cohort, partnered patients had slightly longer dialysis sessions on average than those without a partner, but this difference was not statistically significant. Among partnered patients who completed the SSS, no significant correlation was found between session length and SSS scores, suggesting that session duration does not directly influence self-reported sexual satisfaction in this group.

Although dialysis session length is an important determinant of treatment burden and may influence social functioning and fatigue—longer sessions potentially reducing time available for leisure and interpersonal activities—sexual satisfaction itself appears relatively insensitive to modest differences in treatment duration. This interpretation is consistent with prior work comparing dialysis modalities with differing time burdens. Studies of nocturnal hemodialysis, for example, have reported mixed effects on sexual health outcomes, with some evidence of benefit in selected subgroups, particularly younger female patients (<60 years), who were less likely to report being bothered by the impact of kidney disease on sex life when treated nocturnally [15,17]. Nevertheless, consistent benefits across broader populations have not been demonstrated.

Our data showed no association between dialysis session duration and sexual satisfaction, and this null finding is supported by heterogeneous evidence from both trials and observational studies examining time-intensive regimens versus conventional therapy [15]. Importantly, these discrepancies across studies may reflect differences in patient populations, cultural contexts, and methodological approaches. Furthermore, it is increasingly recognized that subjective sexual well-being in dialysis patients is shaped more by modifiable clinical and psychosocial factors—such as volume status, anemia, depression, and medication effects—than by session duration alone. From a holistic care perspective, while session length may influence daily functioning and psychosocial adjustment, interventions aiming to improve sexual well-being should prioritize addressing these underlying drivers rather than focusing exclusively on treatment time in the dialysis unit.

### 4.9. Sexual Satisfaction and a History of Kidney Transplant

Comparison of SSS scores between dialysis patients with and without a history of kidney transplant yielded a statistically significant result, indicating greater dissatisfaction in patients without transplant experience. This suggests that prior transplantation is associated with more favorable perceptions of sexual life, while patients who have never received a transplant report significantly higher levels of dissatisfaction [6,7,8,9]. Transplantation history was associated with greater satisfaction, paralleling studies showing improved desire and normalized overall satisfaction after kidney transplantation [19,20,34,35]. Patients after successful kidney transplantation tend to report better sexual satisfaction. Evidence shows that renal transplantation restores hormonal balance, improves erectile and desire functions, and leads to higher satisfaction levels, particularly in nondiabetic male recipients [19,35,36]. Female transplant recipients also report significantly better sexual relationships and reduced sexual fear compared with hemodialysis patients [20,34]. These improvements reflect combined physiological recovery and psychosocial relief from treatment burden [34,37].

Potential mechanisms underlying this effect include physiological recovery (improved endocrine balance, reduced uremia, correction of anemia), psychological relief from the burden of chronic dialysis, enhanced autonomy, and a positive shift in body image. Additionally, improved energy levels and reduced treatment fatigue may strengthen relationship intimacy [6,7,8,9,19,20,35,36]. These interconnected biological, psychological, and relational domains likely contribute to the higher sexual satisfaction observed in transplant recipients. Reported gains include better sexual desire, improved orgasmic function, greater intercourse satisfaction, more favorable hormonal milieu, and enhanced body image [6,7,8,9]. Observational series and systematic syntheses consistently document these benefits across domains of male and female sexual function, including desire, arousal, erectile and orgasmic function, and dyspareunia [6,7,8,9]. Reviews focusing on erectile dysfunction after transplantation further highlight meaningful recovery in many patients, while also noting persistent risks associated with cardiometabolic comorbidities and immunosuppressive therapy [10,28,29,33]. Numerous studies have demonstrated that renal transplantation tends to normalize overall sexual satisfaction and improve sexual desire, especially in nondiabetic male recipients [19]. Furthermore, both male and female transplant recipients experience enhanced sexual relationships, increased sexual frequency, and reduced sexual fear compared to patients remaining on hemodialysis [20]. Females after transplantation report significantly better sexual relationship scores and fewer limitations in sexual activity than those on dialysis [20,34]. These improvements likely reflect the multifactorial recovery following transplantation: restoration of endocrine function, correction of anemia, improved nutrition, normalization of testosterone and estrogen levels, and overall enhancement in body image and vitality [34,36]. In men, kidney transplantation has been shown to restore normal sex hormone levels and improve erectile function [36]. Among women, post-transplant hormonal balance correlates with increased sexual desire, though not always with parallel improvements in frequency of sexual activity or global satisfaction [35].

Despite these benefits, sexual dysfunction remains prevalent even among transplant recipients, particularly in individuals with comorbidities or persistent mood symptoms [9,19,23]. This paradox highlights the need to differentiate sexual function from sexual satisfaction as distinct yet interrelated constructs. Moreover, the psychosocial context of transplantation should not be underestimated. Studies confirm that depression, anxiety, and reduced self-esteem remain strong negative correlates of sexual functioning in chronic kidney disease and post-transplant populations [21,25]. Conversely, the sense of regained independence, reduced treatment burden, and improvement in overall well-being after transplantation may enhance sexual self-image and relationship intimacy [37].

In our study, transplant recipients in relationships reported significantly lower SSS scores (reflecting less dissatisfaction) than partnered patients without transplant history. Although our design is cross-sectional and does not allow for causal inference, the direction and magnitude of this association are consistent with the broader body of evidence. Taken together, these findings reinforce the role of transplantation not only in restoring physiological function but also in improving subjective aspects of sexual health, while highlighting the ongoing importance of managing residual risk factors that may attenuate these benefits. From a clinical standpoint, our statistically significant finding underlines the relevance of incorporating sexual health assessment into the long-term follow-up of renal transplant recipients. These results also support the patient-centered approach emphasized in the recent literature [30,31], where healthcare providers are encouraged to address individual priorities and quality-of-life domains, including sexuality, as part of comprehensive post-transplant care.

### 4.10. Sexual Satisfaction and Future Transplant Desire

In our cohort, willingness to undergo future kidney transplantation was not associated with sexual satisfaction as measured by the SSS. This suggests that present-time sexual well-being among dialysis patients may be more strongly determined by current physiological, psychological, and relational factors than by hypothetical expectations of future therapy. While renal transplantation is well documented to improve sexual function and body image across multiple domains [6,7,8,9], our findings indicate that such prospective considerations do not appear to shape current subjective satisfaction.

The absence of a significant relationship may reflect the primacy of immediate factors such as symptom burden, endocrine milieu, mood, and relationship quality in influencing sexual well-being. Patient-reported outcomes research and consensus recommendations similarly emphasize that satisfaction is largely driven by current health status and psychosocial context rather than anticipated treatment trajectories [13,29]. Although our cross-sectional design cannot fully address causality, these findings highlight the need for further research in larger transplant-willing cohorts to clarify whether expectations about future transplantation exert any measurable influence on sexual satisfaction.

### 4.11. Sexual Satisfaction and Urea Levels

There was no statistically significant difference in mean pre-dialysis urea levels between partnered and non-partnered patients. Within the partnered subgroup, the correlation between SSS and pre-dialysis urea was weak and not statistically significant. Although numerically higher urea levels were associated with greater dissatisfaction, this relationship did not reach statistical significance. Overall, no significant association was found between pre-dialysis urea and sexual satisfaction, nor did urea levels differ significantly by partner status, suggesting that broader biopsychosocial influences outweigh the impact of biochemical parameters in this context.

Dialysis adequacy markers, including urea, have demonstrated inconsistent associations with sexual function in the literature. Some studies report clearer relationships, particularly in male patients, while others find no direct link, especially among females [1,4,11,15]. Beyond sexual outcomes, adequacy has been associated with domains such as visual impairment and overall symptom burden in hemodialysis cohorts [11]. This heterogeneity underscores the multifactorial pathophysiology underlying sexual health in end-stage renal disease, encompassing hormonal, vascular, psychological, and comorbid contributors [1,4].

In our study, pre-dialysis urea showed no significant association with SSS, a finding consistent with prior reports demonstrating contradictory links between small-solute clearance and sexual function. Guideline statements likewise caution against assuming that adequacy indices translate directly into sexual satisfaction, which is inherently multidimensional and influenced by endocrine, vascular, neurologic, and psychological factors [15,16]. Taken together, these observations emphasize that sexual satisfaction cannot be reliably predicted from urea levels alone and should be addressed in a broader biopsychosocial framework.

### 4.12. Sexual Satisfaction and Ultrafiltration Volume

In partnered patients, a weak, non-significant negative correlation was observed between SSS and ultrafiltration volume. Numerically, higher ultrafiltration volumes were associated with better sexual satisfaction (lower SSS), and patients with a primary partner tended to have higher ultrafiltration volumes overall. These patterns may reflect more effective volume management, stronger psychosocial support, or better treatment adherence, although causality cannot be inferred. Sexual dysfunction is well documented in hemodialysis, yet the role of dialysis-related parameters such as ultrafiltration in shaping subjective sexual satisfaction remains incompletely understood [1,2,5].

Hydration and volume status plausibly affect sexual health via multiple physiological and psychosocial pathways. Endocrine alterations, vascular function, and mood disturbances are well-recognized mediators in the broader literature on sexual dysfunction in dialysis [1]. In addition, hemodynamic stability, sleep quality, and fatigue represent plausible mechanisms through which ultrafiltration could influence intimacy and satisfaction. Although the association in our study did not reach statistical significance, the observed directionality warrants attention.

Consensus reports emphasize that improving sexual well-being in dialysis requires integrated psychosocial and medical approaches, combining relational and emotional support with optimization of modifiable clinical factors such as volume status, anemia, mineral bone disease, and medication effects [23,25]. In this context, our findings support the need for future longitudinal and interventional studies to clarify potential causal mechanisms, assess whether targeted volume management could contribute to improvements in sexual well-being, and explore the synergistic role of psychosocial care in maximizing these outcomes.

### 4.13. Sexual Satisfaction and Kt/V

Kt/V is a core parameter of dialysis adequacy, with guideline targets generally aiming for ≥1.3–1.4 in thrice-weekly hemodialysis. Evidence linking adequacy to sexual function has been mixed: some preliminary studies, particularly in male patients, have suggested associations, while others—especially in female cohorts—have not demonstrated a clear relationship [16,23]. Our findings add to this heterogeneous picture.

In our study, there was no statistically significant difference in dialysis adequacy (Kt/V) between patients with and without a partner. Among partnered patients, the correlation between Kt/V and SSS was weak and not statistically significant. Numerically, there was a non-significant trend suggesting that higher adequacy might be associated with slightly lower dissatisfaction, but the association was weak and inconclusive.

These results align with prior reports showing limited direct coupling between adequacy metrics and subjective sexual satisfaction in cross-sectional analyses [16]. While selected studies in men have indicated possible improvements in sexual function with higher adequacy, such effects remain inconsistent and are likely influenced by confounding factors such as comorbidity burden, systemic inflammation, and depression [16]. Overall, our null findings underscore that dialysis adequacy, as measured by Kt/V, may be necessary for overall health but is not sufficient on its own to explain variations in sexual satisfaction. Broader physiological, psychological, and relational determinants appear to exert stronger influence in this domain.

### 4.14. Sexual Satisfaction and Urea Reduction Ratio

In partnered patients, the correlation between urea reduction ratio and SSS was weak and non-significant. There was also no statistically significant difference in the urea reduction ratio between patients with and without a partner. Although a weak negative trend was observed—whereby a higher urea reduction ratio was numerically associated with slightly better sexual satisfaction (lower SSS)—this relationship did not reach statistical significance.

As with Kt/V, the urea reduction ratio represents a core dialysis adequacy index, but its relationship with subjective sexual satisfaction has been inconsistent across studies. Our findings parallel prior work showing limited or absent direct associations between adequacy metrics and patient-reported sexual outcomes, despite the clear importance of adequacy for other clinical domains and complications, including visual function and overall symptom burden [11,16].

The absence of a robust association in our cohort reinforces the conclusion that sexual satisfaction in dialysis patients is not a simple function of urea kinetics. Mixed findings in the literature [16,23,25] suggest that while biochemical adequacy is necessary for clinical stability, it does not adequately capture the complex interplay of physiological, psychological, relational, and social factors that determine sexual well-being. A nuanced, biopsychosocial approach, therefore, remains essential for understanding and addressing sexual satisfaction in this population.

### 4.15. Sexual Satisfaction in Dialysis Patients and Healthy Controls

The comparison between dialysis patients and healthy controls revealed no statistically significant differences in mean age or gender distribution, reducing the likelihood of demographic confounding. Importantly, no significant differences in sexual satisfaction scores were found between dialysis patients and healthy controls overall, nor within gender strata (male patients vs. male controls, female patients vs. female controls). Despite very high sexual dysfunction rates reported in hemodialysis—particularly among women (often ~80%) [19,20,21,23,30]—overall satisfaction may remain preserved for many due to adaptation and intimacy beyond intercourse frequency. This aligns with data showing that many women on hemodialysis report being moderately to very satisfied even when sexually inactive [13,28,30], helping explain our similar SSS scores to controls.

To reconcile these findings with the extensive literature on high dysfunction prevalence, we emphasize the conceptual distinction between sexual dysfunction (physiologic impairment) and sexual satisfaction (subjective appraisal). Prior studies show that many dialysis patients—particularly women—fulfill criteria for dysfunction due to low activity levels, yet still report being satisfied with their sexual lives. This suggests adaptation, recalibrated expectations, emotional intimacy, and supportive partner relationships may preserve subjective satisfaction despite physiologic limitations [1,2,3,4,13]. Prior work highlights that many women on hemodialysis report satisfaction despite sexual inactivity [13,23,30]. This context supports our finding of similar SSS scores to controls despite the known physiologic burden. Our results reinforce the critical distinction between physiologic sexual dysfunction and subjective sexual satisfaction. Mor et al. demonstrated that many women on hemodialysis meet dysfunction criteria—often due to sexual inactivity—yet still report satisfactory sexual lives [13]. Such findings highlight that while physiologic limitations are prevalent, patients may adapt expectations and derive satisfaction through other dimensions of intimacy, emotional closeness, and supportive relationships.

The absence of significant differences in SSS between dialysis patients and healthy controls may thus reflect resilience and coping strategies among long-term dialysis patients. Through recalibrated expectations and redefined sources of intimacy, individuals may maintain a sense of satisfaction despite objective health challenges. This interpretation is consistent with prior work showing that satisfaction can remain stable even when dysfunction rates are high, especially when patients derive meaning from intimacy beyond intercourse frequency [13,28,29].

Taken together, our findings argue for a multidimensional approach to sexual health in end-stage renal disease. Patient-reported outcome measures, including subjective satisfaction, should be integrated alongside clinical and physiological assessments to capture the full spectrum of sexual well-being. This perspective underscores the importance of holistic, communication-focused care strategies that address both dysfunction and subjective quality-of-life domains [12]. Integrating sexual health into holistic, patient-centered nephrology care is essential. Multidisciplinary strategies—including psychosocial counseling, patient–partner communication, and pre- or post-transplant sexual health education—should be routinely implemented. These approaches align with global recommendations for person-centered care, emphasizing patient empowerment and shared decision-making in chronic kidney disease management [31].

While pharmacological treatments (e.g., PDE5 inhibitors) can be effective for erectile dysfunction, psychosocial counseling, mood management, and relational therapy remain central for improving overall sexual satisfaction, especially among female patients and those without erectile dysfunction [30,34,38]. The absence of associations between routine hemodialysis metrics and SSS should be interpreted cautiously. Possible explanations include limited power to detect small effects, the measurement scope of the SSS (global satisfaction rather than domain-specific function), and unmeasured psychosocial confounders such as depression, anxiety, relationship quality, and body image—factors known to influence sexual outcomes in hemodialysis, particularly among women and patients with diabetes or dyslipidemia [9,19,20,21,23,30,34,35,38].

Studies demonstrate that sexual functioning correlates negatively with depressive and anxiety symptoms, and that satisfaction with sexual life is inversely related to general mental health indices [25]. Therefore, addressing mood and anxiety disorders may substantially improve perceived sexual satisfaction. Clinicians should devote greater attention to sexual health in routine nephrology care. An emphasis on patient-centered care—where treatment decisions reflect individual priorities and quality-of-life goals—is increasingly recognized worldwide [31,39]. Healthcare providers should discuss sexual concerns openly, explore whether patients perceive sexual inactivity as a problem, and tailor management accordingly [13,31]. As dialysis is a lifelong therapy, addressing sexuality forms part of comprehensive psychosocial support [4,30,40].

In conclusion, sexual dissatisfaction is common among hemodialysis patients but appears largely independent of routine dialysis measures. It coexists with high rates of sexual dysfunction driven by comorbidities such as diabetes, dyslipidemia, and depression [19,21,23,25]. Kidney transplantation appears to normalize desire and satisfaction [19,20,34,35], further supporting multidisciplinary, patient-centered approaches to care [30,31,41]. Future longitudinal research should explore causal pathways and evaluate targeted interventions aimed at improving both sexual function and subjective satisfaction [30,42].

### 4.16. Potential Sources of Bias

Selection bias is possible despite broad age/sex matching with controls; participation may be enriched for individuals more comfortable discussing sexuality. Information/response bias may arise from self-report and social desirability, even with private administration. Recall bias is mitigated but not eliminated by a 4-week recall window. Confounding remains a key concern—unmeasured diabetes, dyslipidemia, depression/anxiety, and medication effects can influence sexual outcomes [19,21,23,25,38]. Cultural bias may also reduce disclosure in sociocultural contexts where sexuality is stigmatized, particularly for women [23,30]. These factors could attenuate detectable associations between hemodialysis metrics and satisfaction.

### 4.17. Limitations and Perspectives for Future Research

This study has several limitations that should be acknowledged. First, the sample size (72 patients and 52 controls) limits statistical power, especially in subgroup analyses such as education level or vascular access. Small effect sizes may therefore remain undetected. Moreover, recruitment during routine dialysis sessions may introduce selection bias, as individuals more comfortable discussing sexuality may be more likely to participate. Second, the cross-sectional design prevents conclusions about causality or the direction of observed associations. Third, sexual satisfaction was assessed with the SSS questionnaire, an instrument that captures general aspects of satisfaction but may not fully reflect the complexity of sexual function and intimacy in hemodialysis patients. Social, psychological, cultural, and relational factors, likely important contributors to sexual satisfaction, were not systematically evaluated. Fourth, self-reported measures are subject to reporting bias, particularly given the sensitive nature of the topic. Fifth, sociocultural factors, including reticence to discuss sexual matters, may have contributed to underreporting of dissatisfaction, as observed in prior cross-cultural studies of chronic kidney disease-related sexual health [4,23,30].

Despite these limitations, the findings highlight important directions for future research. Larger, multicenter studies are warranted to confirm the observed associations, especially regarding the impact of kidney transplantation on sexual satisfaction. Longitudinal designs could clarify temporal relationships between dialysis duration, treatment parameters, and changes in sexual well-being. Furthermore, qualitative and mixed-methods studies may provide richer insights into patient perspectives and unmet needs. Finally, interventions aimed at improving sexual health in dialysis populations, whether psychosocial counseling, medical management, or multidisciplinary support, should be systematically developed and evaluated.

Future longitudinal studies are planned to evaluate temporal changes in sexual satisfaction, particularly before and after renal transplantation, to clarify causal relationships and adaptation over time.

## 5. Conclusions

In this cohort of hemodialysis patients, sexual dissatisfaction was common but was not significantly associated with most demographic, clinical, or dialysis-related parameters. Age, gender, marital status, educational attainment, dialysis shift, vascular access type, dialysis adequacy indices (Kt/V, urea reduction ratio, pre-dialysis urea), dialysis session duration, and ultrafiltration volumes showed no consistent or statistically significant associations with SSS scores.

A history of kidney transplantation was the only factor significantly related to sexual satisfaction: patients with prior transplantation reported lower SSS scores, reflecting less dissatisfaction, compared with those without a transplant history. Dialysis duration and ultrafiltration volume were higher in patients with a partner than in those without, although these did not correlate with SSS outcomes.

Comparison with healthy controls revealed no significant differences in sexual satisfaction scores, age, or gender distribution, suggesting that dialysis patients do not differ markedly from the general population in reported sexual satisfaction when assessed using the SSS.

Taken together, these findings indicate that while sexual dissatisfaction is prevalent among hemodialysis patients, it appears largely independent of routine demographic or dialysis-related measures, with kidney transplantation history emerging as the most relevant clinical factor. Routine nephrology care should explicitly include sexual health screening and counseling. Multidisciplinary pathways (nephrology, psychology/psychiatry, sexual health, nursing, social work) should address depression/anxiety, metabolic risk, medication effects, hormonal status, and relationship factors.

## Figures and Tables

**Table 1:** Sexual Satisfaction Scale scores by age.

	n	Age (Years) Mean, SD (Range)	SSS Mean, SD (Range)	Pearson’s Correlation SSS-Age	*p* “With” vs. “Without” Partner
with partner	54	60.94, 11.23 (29–81)	14.0, 6.5 (4–24)	r = 0.249 *p* = 0.069	0.100
without partner	18	67.44, 14.77 (28–84)	0		

SSS—Sexual Satisfaction Scale.

**Table 2:** Sexual Satisfaction Scale scores by gender.

	Male	Female	*p*
with partner (n) SSS mean, SD (range)	34 13.53, 7.74 (4–24)	20 10.79, 7.57 (4–24)	0.220
without partner (n)	10	8	

SSS—Sexual Satisfaction Scale.

**Table 3:** Sexual Satisfaction Scale scores by marital status distribution.

	Married	Single	Widowed	*p*
with partner (n) SSS mean, SD (range)	50 12.52, 7.76 (4–24)	4 11.00, 9.02 (4–24)	0	0.711
without partner (n)	0	8	10	

SSS—Sexual Satisfaction Scale.

**Table 4:** Sexual Satisfaction Scale scores by education level.

	Primary Education	Secondary Education	Higher Education	*p*
with partner (n) SSS mean, sd (range)	19 11.91, 9.53 (4–24)	21 11.27, 5.08 (7–24)	13 15.75, SD = 7.30 (5–24)	0.633
without partner (n)	5	8	4	

SSS—Sexual Satisfaction Scale.

**Table 5:** Sexual Satisfaction Scale scores by dialysis duration.

	n	Dialysis Duration (Months) Mean, SD (Range)	SSS Mean, SD (Range)	Pearson’s Correlation SSS-Dialysis Duration	*p* “With” vs. “Without” Partner
with partner	54	60.24, 71.94 (1–317)	14.0, 6.5 (4–24)	r = −0.11 *p* = 0.44	0.011
without partner	18	28.00, 28.73 (2–108)	0		

SSS—Sexual Satisfaction Scale.

**Table 6:** Sexual Satisfaction Scale scores by vascular access for dialysis.

	Arterio-Venous Fistula	Central Venous Catheter	*p*
with partner (n) SSS mean, SD (range)	46 11.92, 7.46 (4–24)	8 11.88, 6.47 (5–24)	0.991
without partner (n)	15	3	

AVF—arteriovenous fistula, CVC—central venous catheter, SSS—Sexual Satisfaction Scale.

**Table 7:** Sexual Satisfaction Scale scores by dialysis shift.

	1st Dialysis Shift	2nd Dialysis Shift	3rd Dialysis Shift	*p*
with partner (n) SSS mean, SD (range)	19 12.95, 9.19 (4–24)	20 12.25, 6.78 (4–24)	15 11.93, 7.57 (4–24)	0.928
without partner (n)	8	7	3	

SSS—Sexual Satisfaction Scale.

**Table 8:** Sexual Satisfaction Scale scores by duration of dialysis session.

	n	Duration of Dialysis Session (Hours) Mean, SD (Range)	SSS Mean, SD (Range)	Pearson’s Correlation SSS-Duration of Dialysis Session	*p* “With” vs. “Without” Partner
with partner	54	4.04, SD = 0.44 (2.0–5.0)	14.0, 6.5 (4–24)	r = 0.047 *p* = 0.739	0.331
without partner	18	3.92, SD = 0.46 (3.0–5.0)	0		

SSS—Sexual Satisfaction Scale.

**Table 9:** Sexual Satisfaction Scale scores by history of kidney transplant.

	n	Age (Years) Mean, SD (Range)	SSS Mean, SD (Range)	Pearson’s Correlation SSS-Age	*p* “With” vs. “Without” Partner
with partner	54	60.94, 11.23 (29–81)	14.0, 6.5 (4–24)	r = 0.249 *p* = 0.069	0.100
without partner	18	67.44, 14.77 (28–84)	0		

SSS—Sexual Satisfaction Scale.

**Table 10:** Sexual Satisfaction Scale scores by future transplant desire.

	Future Transplant Desire	No Future Transplant Desire	*p*
with partner (n) SSS mean, SD (range)	5 11.50, 4.93 (6–18)	49 14.14, 7.02 (4–24)	0.587
without partner (n)	2	16	

SSS—Sexual Satisfaction Scale.

**Table 11:** Sexual Satisfaction Scale scores by urea levels.

	n	Urea Level (mg/dL) Mean, SD (Range)	SSS Mean, SD (Range)	Pearson’s Correlation SSS-Urea Level	*p* “With” vs. “Without” Partner
with partner	54	120.89, SD = 37.42 (72–202)	14.0, 6.5 (4–24)	r = 0.185 *p* = 0.189	0.138
without partner	18	136.19, SD = 34.51 (72–191)	0		

SSS—Sexual Satisfaction Scale.

**Table 12:** Sexual Satisfaction Scale scores by ultrafiltration volume.

	n	Ultrafiltration (mL) Mean, SD (Range)	SSS Mean, SD (Range)	Pearson’s Correlation SSS-Dialysis Duration	*p* “With” vs. “Without” Partner
with partner	54	2438, SD = 947 (300–4200)	14.0, 6.5 (4–24)	r = −0.183 *p* = 0.195	0.0029
without partner	18	2438, SD = 947 (300–4200)	0		

SSS—Sexual Satisfaction Scale.

**Table 13:** Sexual Satisfaction Scale scores by Kt/V.

	n	Kt/V Mean, SD (Range)	SSS Mean, SD (Range)	Pearson’s Correlation SSS-Dialysis Duration	*p* “With” vs. “Without” Partner
with partner	54	1.297, SD = 0.264 (0.661–1.895)	14.0, 6.5 (4–24)	r = −0.240 *p* = 0.195	0.702
without partner	18	1.343, SD = 0.489 (0.818–1.572)	0		

SSS—Sexual Satisfaction Scale.

**Table 14:** Sexual Satisfaction Scale scores and urea reduction ratio.

	n	URR Mean, SD (Range)	SSS Mean, SD (Range)	Pearson’s Correlation SSS-Dialysis Duration	*p* “With” vs. “Without” Partner
with partner	54	0.659, SD = 0.075 (0.420–0.802)	14.0, 6.5 (4–24)	r = −0.14 *p* = 0.25	0.76
without partner	18	0.666, SD = 0.089 (0.507–0.904)	0		

URR—urea reduction ratio, SSS—Sexual Satisfaction Scale.

**Table 15:** Age, gender, and Sexual Satisfaction Scale scores in dialysis patients and healthy controls.

	Dialysis Patients	Healthy Controls	*p*
n mean age (years), SD (range)	72 62.3, 12.6 (28–84)	52 60.6, 13.4 (36–86)	0.268
n male/female	72 44/28	52 32/20	1.00
n SSS mean score (SD)	72 9.18 (8.64)	52 9.26 (6.25)	0.951

SSS—Sexual Satisfaction Scale.

**Table 16:** Sexual Satisfaction Scale scores by gender in dialysis patients and healthy controls.

		n	SSS Mean (SD)	*p*
males	dialysis	44	10.45 (8.95)	0.395
	healthy	20	8.84 (5.72)	
females	dialysis	28	7.50 (7.92)	0.318
	healthy	32	9.44 (6.82)	

SSS—Sexual Satisfaction Scale.

## Data Availability

The data presented in this study are available on request from the corresponding author. The data are not publicly available due to privacy restrictions.

## Abbreviations

The following abbreviations are used in this manuscript:

SD	Standard deviation
SSS	Sexual Satisfaction Scale
URR	Urea reduction ratio
AVF	Arteriovenous fistula
CVC	central venous catheter

## References

[1] Chou J., Kiebalo T., Jagiello P., Pawlaczyk K. (2021). Multifaceted Sexual Dysfunction in Dialyzing Men and Women: Pathophysiology, Diagnostics, and Therapeutics. Life.

[2] Filocamo M.T., Zanazzi M., Li Marzi V., Lombardi G., Del Popolo G., Mancini G., Salvadori M., Nicita G. (2009). Sexual dysfunction in women during dialysis and after renal transplantation. J. Sex Med..

[3] Álvarez-Villarreal M., Velarde-García J.F., Chocarro-Gonzalez L., Pérez-Corrales J., Gueita-Rodriguez J., Palacios-Ceña D. (2019). Body Changes and Decreased Sexual Drive after Dialysis: A Qualitative Study on the Experiences of Women at an Ambulatory Dialysis Unit in Spain. Int. J. Environ. Res. Public Health.

[4] Abdul Rahman N., Ghani M., Kausar S., Sadiqa A., Khalid A. (2023). Revealing the Connection Between Hemodialysis and Sexual Physiology in Women with End-Stage Renal Disease. Cureus.

[5] Beal-Lloyd D., Groh C.J. (2012). Dialysis and sexuality. Nephrol. Nurs. J..

[6] Agarwal K.A., Pavlakis M. (2021). Sexuality, Contraception, and Pregnancy in Kidney Transplantation. Kidney Med..

[7] Barroso L.V., Miranda E.P., Cruz N.I., Medeiros M.A., Araújo A.C., Mota Filho F.H., Medeiros F.C. (2008). Analysis of sexual function in kidney transplanted men. Transpl. Proc..

[8] Pertuz W., Castaneda D.A., Rincon O., Lozano E. (2014). Sexual dysfunction in patients with chronic renal disease: Does it improve with renal transplantation?. Transpl. Proc..

[9] Vranješ I.M., Školka I., Jakab J., Krajina I., Krajina V., Šantić A., Zibar L. (2022). Sexual function in hemodialysis and post-renal transplant women in a relationship: A cross-sectional study. Int. Urol. Nephrol..

[10] Miron A., Nistor I., Moroșanu C., Sirițeanu L., Pricop C., Puia D., Covic A. (2025). Prevalence, risk factors, and severity of erectile dysfunction following renal transplantation. Int. Urol. Nephrol..

[11] Sułkowski L., Rubinkiewicz M., Matyja A., Matyja M. (2023). Visual Impairment in Hemodialyzed Patients-An IVIS Study. Medicina.

[12] Sułkowski L., Matyja A., Matyja M. (2024). Social Support and Quality of Life in Hemodialysis Patients: A Comparative Study with Healthy Controls. Medicina.

[13] Mor M.K., Sevick M.A., Shields A.M., Green J.A., Palevsky P.M., Arnold R.M., Fine M.J., Weisbord S.D. (2014). Sexual function, activity, and satisfaction among women receiving maintenance hemodialysis. Clin. J. Am. Soc. Nephrol..

[14] Nowrooz S., Alanazi T., Al-Ghamdi A., Alzahrani A., Alshammari A., AlYaqoot N., Almutraid M., Jaradat A., El-Agroudy A. (2023). Quality of Life among Hemodialysis Patients: Role of the Dialysis Shift. Saudi J. Kidney Dis. Transpl..

[15] Bass A., Ahmed S.B., Klarenbach S., Culleton B., Hemmelgarn B.R., Manns B. (2012). The impact of nocturnal hemodialysis on sexual function. BMC Nephrol..

[16] Kim J.H., Doo S.W., Yang W.J., Kwon S.H., Song E.S., Lee H.J., Lim I.S., Hwang H., Song Y.S. (2014). Association between the hemodialysis adequacy and sexual dysfunction in chronic renal failure: A preliminary study. BMC Urol..

[17] Sułkowski L., Matyja A., Matyja M. (2025). The Impact of Dialysis Duration on Multidimensional Health Outcomes: A Cross-Sectional Study. J. Clin. Med..

[18] Keskin G., Babacan Gümüş A., Taşdemir Yiğitoğlu G. (2019). Sexual dysfunctions and related variables with sexual function in patients who undergo dialysis for chronic renal failure. J. Clin. Nurs..

[19] Al Khallaf H.H. (2010). Analysis of sexual functions in male nondiabetic hemodialysis patients and renal transplant recipients. Transpl Int..

[20] Noohi S., Azar M., Behzadi A.H., Barbati M.E., Haghshenas A., Amoozgar B., Karami M. (2010). Comparison of sexual function in females receiving haemodialysis and after renal transplantation. J. Ren. Care.

[21] Peng Y.S., Chiang C.K., Kao T.W., Hung K.Y., Lu C.S., Chiang S.S., Yang C.S., Huang Y.C., Wu K.D., Wu M.S. (2005). Sexual dysfunction in female hemodialysis patients: A multicenter study. Kidney Int..

[22] Kılıç E.K., Kılıç İ., Görgülü Y., Üstündağ S. (2024). Quality of life and sleep, depression, family functioning, and marital adjustment in patients on hemodialysis and peritoneal dialysis. Ther. Apher. Dial..

[23] Saglimbene V., Natale P., Palmer S., Scardapane M., Craig J.C., Ruospo M., Gargano L., Lucisano G., Török M., Celia E. (2017). The prevalence and correlates of low sexual functioning in women on hemodialysis: A multinational, cross-sectional study. PLoS ONE.

[24] Savadi H., Khaki M., Javnbakht M., Pourrafiee H. (2016). The Impact of Hemodialysis on Sexual Function in Male Patients using the International Index of Erectile Function Questionnaire (IIEF). Electron Physician.

[25] Theofilou P.A. (2012). Sexual functioning in chronic kidney disease: The association with depression and anxiety. Hemodial. Int..

[26] Ritvo P.G., Fischer J.S., Miller D.M., Andrews H., Paty D.W., LaRocca N.G. (1997). Multiple Sclerosis Quality of Life Inventory (MSQLI): User’s Manual.

[27] Stewart A.L., Ware J.E. (1992). Measuring Functioning and Well-Being: The Medical Outcomes Study Approach.

[28] Vecchio M., Palmer S., De Berardis G., Craig J., Lucisano G., Johnson D., Pellegrini F., Nicolucci A., Sciancalepore M., Saglimbene V. (2012). Sexual dysfunction in women with ESRD requiring hemodialysis. Clin. J. Am. Soc. Nephrol..

[29] Pyrgidis N., Mykoniatis I., Tishukov M., Sokolakis I., Nigdelis M.P., Sountoulides P., Hatzichristodoulou G., Hatzichristou D. (2021). Sexual Dysfunction in Women with End-Stage Renal Disease: A Systematic Review and Meta-Analysis. J. Sex Med..

[30] Corbett K.S., Chang D.H., Riehl-Tonn V.J., Ahmed S.B., Rao N., Kamar F., Dumanski S.M. (2024). Sexual Activity, Function, and Satisfaction in Reproductive-Aged Females Living with Chronic Kidney Disease. Healthcare.

[31] Finkelstein F.O., Finkelstein S.H. (2014). Sexual Inactivity among hemodialysis patients: The patients’ perspective. Clin. J. Am. Soc. Nephrol..

[32] Oyekçin D.G., Gülpek D., Sahin E.M., Mete L. (2012). Depression, anxiety, body image, sexual functioning, and dyadic adjustment associated with dialysis type in chronic renal failure. Int. J. Psychiatry Med..

[33] Soykan A., Boztas H., Kutlay S., Ince E., Nergizoglu G., Dileköz A.Y., Berksun O. (2005). Do sexual dysfunctions get better during dialysis? Results of a six-month prospective follow-up study from Turkey. Int. J. Impot. Res..

[34] Basok E.K., Atsu N., Rifaioglu M.M., Kantarci G., Yildirim A., Tokuc R. (2009). Assessment of female sexual function and quality of life in predialysis, peritoneal dialysis, hemodialysis, and renal transplant patients. Int. Urol. Nephrol..

[35] Ghazizadeh S., Lessan-Pezeshki M. (2007). Reproduction in women with end-stage renal disease and effect of kidney transplantation. Iran J. Kidney Dis..

[36] El Hennawy H.M., Safar O., Al Faifi A.S., Shalkamy O., El Madawie M.Z., Thamer S., Almurayyi M., Alqarni A.M., Amri S.S., Hawan A.A. (2024). Kidney transplantation restores sex hormone profile and improves sexual function in ESRD patients with erectile dysfunction. Arch Ital. Urol. Androl..

[37] Mitema D., Jaar B.G. (2016). How Can We Improve the Quality of Life of Dialysis Patients?. Semin Dial..

[38] Fugl-Meyer K.S., Nilsson M., Hylander B., Lehtihet M. (2017). Sexual Function and Testosterone Level in Men with Conservatively Treated Chronic Kidney Disease. Am. J. Mens. Health.

[39] Avramovic M., Stefanovic V. (2012). Health-related quality of life in different stages of renal failure. Artif. Organs..

[40] Zeighami S., Dehghankhalili S., Heiran K., Azarchehry S.P., Heiran A., Sayadi M., Azadian F. (2022). Comparison of Male and Female Sexual Dysfunction between Hemodialysis and Peritoneal Dialysis in Patients with End-Stage Renal Disease: An Analytical Cross-Sectional Study. Int. J. Endocrinol..

[41] Perri A., Ilacqua A., Valenti M., Aversa A. (2018). Effects of nutraceuticals on sexual satisfaction and lower urinary tract symptoms in a cohort of young-old men. Phytother. Res..

[42] Toorians A.W., Janssen E., Laan E., Gooren L.J., Giltay E.J., Oe P.L., Donker A.J., Everaerd W. (1997). Chronic renal failure and sexual functioning: Clinical status versus objectively assessed sexual response. Nephrol. Dial. Transpl..

